# Immune Response to Combination Therapy for Non-Hodgkin Lymphomas

**DOI:** 10.1371/journal.pone.0081672

**Published:** 2013-12-06

**Authors:** Robert F. Weiss, Merlin G. Miller, John F. Cronin

**Affiliations:** 1 President, Back Bay Biosciences LLC, Boston, Massachusetts, United States of America; 2 Consultant, Back Bay Biosciences LLC, Boston, Massachusetts, United States of America; 3 Physical Sciences Inc., Andover, Massachusetts, United States of America; Mie University Graduate School of Medicine, Japan

## Abstract

A parametric model of tumor response to combination therapy in the presence of an immune system is described. Synergistic mechanisms which induce tumor regression are simulated with a coupled set of equations. The simulations are first compared to tumor history data obtained with a SCID mouse model to determine key parameters; predictions are then made for an immune-competent animal. The minimum immune cell birth rate relative to malignant B-cell birth rate necessary to induce tumor regression is determined, and optimization of drug combinations in the presence of an immune response is explored. The delayed effect of an immune response relative to drug scheduling is examined, and a mechanism for disease transformation in heterogeneous tumors is proposed.

## Introduction

In a recent study [1], we developed a parametric model of tumor growth in B-cell lymphomas to ***predict*** the quantitative effects of combination drug therapy on malignant cell population dynamics. Our model extends earlier theoretical studies of immune response to tumor growth [2], [3] by deriving values of key rate constants from mono-therapy experiments that are then utilized to make combination therapy predictions. Our model is directed exclusively at advanced treatment of B-cell lymphomas. We showed that the combined effects of pro-apoptotic (as-bcl-2) and direct kill (anti-CD-20) mechanisms were synergistic, amplifying the micro-environmental acceleration of cell death rates. The key parameters were determined by sequential analysis of data taken with Severe Combined Immune-Deficient (SCID) mouse experiments in which each drug was *separately administered*. Tumor growth was then successfully predicted for the *combination* therapy at early times (0 to 7 days). Modification of a key parameter resulted in the successful prediction of late time SCID data as well.

We have since refined our model in several respects. First, we have added a description of the animal's immune response to the presence of malignant cell antigen, and the multiple roles of anti-CD-20 in amplifying this response. This is particularly important, as the behavior of individual or combination drug therapies can behave very differently in immune competent animals and humans. Second, we have permitted the key parameters to reflect the presence or depletion of drugs in the animal, so as to ultimately model the effects of drug dosage and treatment scheduling. Third, we allow for the presence of more aggressive clones that are at first unobservable, but capable of transforming the disease state from “indolent” to “aggressive.”

We will first describe the immune response model in terms of the separate biological mechanisms it simulates. We then apply this model to the cases previously studied, primarily as an illustration of the influence of an immune response with combination immunotherapy. A very brief review of the SCID data analysis previously reported [1] is provided, as we are focusing on the predicted effects of combination immunotherapy in an *immune-competent* mouse, rather than further statistical analysis of the published SCID data. Finally, we illustrate the application of our expanded model to optimization of drug combination dosages, drug scheduling, and the issue of indolent to aggressive disease transformation.

## The Model

The previously developed model [1] equated the temporal rate of change of the malignant B-cell population (N_B_) to the sum of terms that characterize the various mechanisms that increase or decrease the population in the absence of an immune response. These mechanisms include the normal B-cell birth rate, the malignant B-cell death rate, and the potential amplification of the malignant population death rate by hypoxia and lack of nutrients in the micro-environment (modeled to first-order by the ratio of the malignant B-cell population to its initial value). We extend this model by adding the immune response of T-cells, the second most important component, after B-cells, of a healthy immune system. This requires the addition of terms in the B-cell population dynamics equation to simulate the environmental, direct, and T-cell-assisted kill mechanisms of anti-CD-20, and a second coupled equation that describes T-cell population (N_T_) dynamics. The coupled set of equations is:
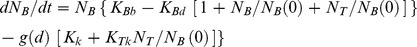
(1a)and
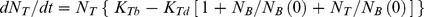
(2a)


As in Ref. 1, it is convenient to non-dimensionalize all cell populations by N_B_(0), the initial B-cell value, and elapsed time by the reciprocal of the B-cell birth rate K_Bb_, resulting in:
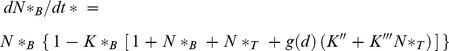
(1b)

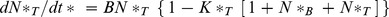
(2b)where

t* = non-dimensional time  = tK_Bb_

N_B_(0) = initial cell population

N*_B_ = B-cell number/N_B_(0)

N*_T_ = T-cell number/N_B_(0)

K*_B_ = B-cell death rate/B-cell birth rate  = K_Bd_/K_Bb_

K*_T_ = T-cell death rate/T-cell birth rate  = K_Td_/K_Tb_

K″ = drug induced B-cell kill rate/B-cell death rate  = K_k_/K_Bd_

g(d) = dependence of kill rate (K_k_) on drug concentration (d)

K″′ = T-cell induced B-cell kill rate/B-cell death rate  = K_Tk_/K_Bd_

B = T-cell birth rate/B-cell birth rate  = K_Tb_/K_Bb_

The first term of each equation is the population growth term. The second term models the overcrowding effects of the micro-environment, where K*_B_ and K*_T_ are, respectively, ratios of malignant B-cell and T-cell death rates to birth rates. When malignant B-cells are treated with pro-apoptotic drugs such as as-bcl-2, we replace K*_B_ by the symbol K′, which is expected to be greater than K*_B_ due to suppression of bcl-2 and a corresponding increase in B-cell death rate. ***Parameter B is the ratio of T-cell to B-cell birth rates***, which will be critical to the immune response. Normal cells have equal birth and death rates in steady state equilibrium. While not essential to the model, if we assume that all normal B-cells and T-cells also have identical birth and death rates, then K*_T_ = 1/B, eliminating one parameter.

The ***direct*** kill effects of anti-CD-20 via penetration of the malignant cell wall are represented by K″g(d), where K″ is the ratio of the direct kill rate to malignant cell death rate and g(d) is a function of drug dosage. The same function of dosage is assumed to apply to the last term in Eq. (1a), which models the ***indirect*** kill of malignant B-cells by T-cells. This mechanism requires malignant B-cell receptors to be recognized by activated T-cells, and should also be dosage-dependent. We note that the g(d) term is omitted from Eq. (2a), as it is assumed that anti-CD-20 has no effect on T-cell populations. Finally, parameter K″′ is the ratio of the ***indirect*** T-cell kill rate to the malignant B-cell death rate. We summarize the definitions of key parameters and the mechanisms modeled by the terms on the right hand side of Eq. 1b, along with typical values of the key parameters, in Table 1.

**Table 1:** Key Parameters and Their Typical Values.

Term in Eq. (1b)	N*_B_	K*_B_ (1+N*_B_ + N*_T_)	K*_B_ g(d) (K″ + K″′N*_T_)
Mechanism	Non-dimensional malignant B-cell population growth	Micro-environment effects on malignant B-cell death rate	Direct kill and T-cell-assisted kill of malignant B-cells
Parameters	1/K_Bb_ = reciprocal of B-cell birth rate	K*_B_ = ratio of B-cell death to birth rates	direct (K″) and indirect (K″′) kill rate ratios
Parameter range	1-10 days	0.05 to 0.5	1 to 10 each

## Baseline Tumor Response

**Figure 1:**
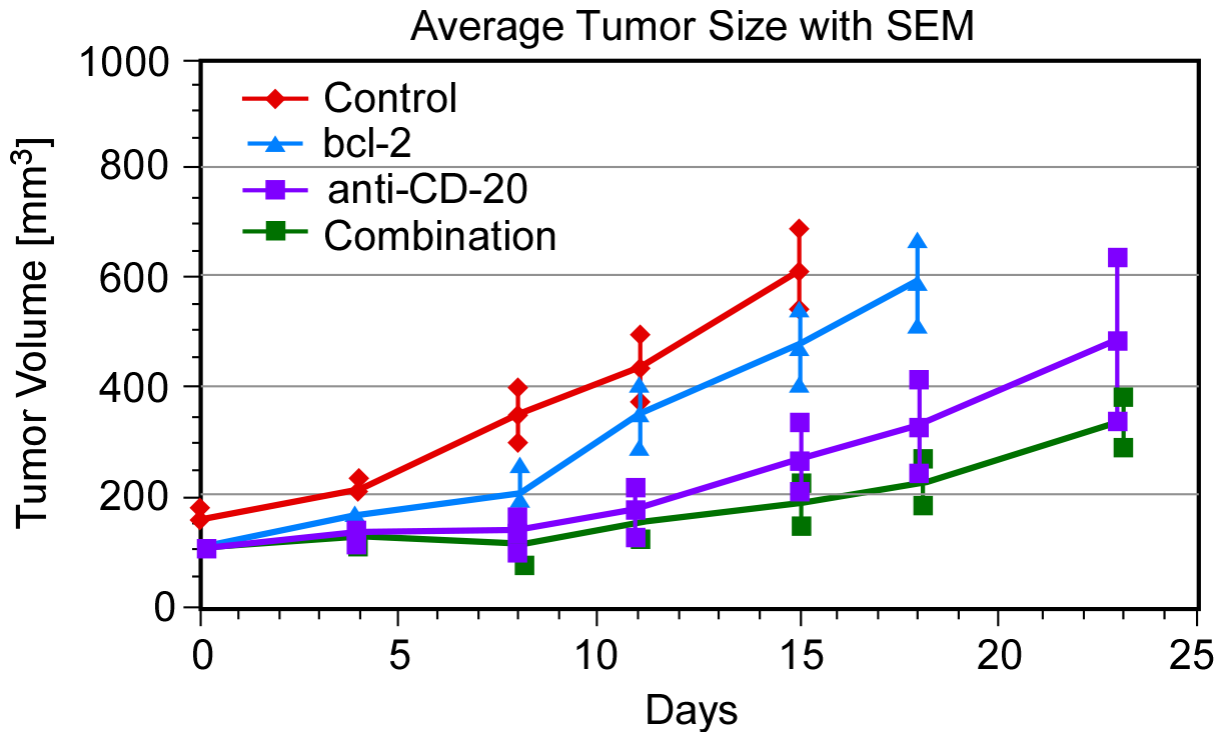
Tumor growth in SCID mice determined by MRI measurements [1].

A summary of the data analyzed in Ref.1 is provided in Figure 1. Tumor volume was measured periodically by taking MRI “slices” and integrating them for each data point. Ten animals were used in each of four series of experiments utilizing:1) mut-as-bcl-2, an oligonucleotide with no effect on a mutated B-cell; the “control”; 2) as-bcl-2, an “anti-sense” pro-apoptotic oligonucleotide designed to increase K*_B_; 3) anti-CD-20 monoclonal antibody (rituximab), which kills cells exhibiting CD-20 via multiple mechanisms; and 4) a combination of as-bcl-2 and rituximab [4], [5], [6]. Both the mean tumor volume histories and their related Standard Error of the Mean (SEM) are shown in Figure 1.

The parameters listed in Table 1 were determined by adjusting them sequentially until the net differences between the model predictions and data over their time histories were minimized. For these SCID mouse experiments, we set N*_T_ = 0 in our model. Each mono-therapy experimental set provided one parameter (i.e., characteristic time 1/K_b_ and K*_B_ from the control set, K′ from the as-bcl-2 experiments, and K″ from the anti-CD-20 set, all for a single dosage such that g(d) was set at 1.0). With these parameters “hard-wired,” we ***predicted*** the tumor volume time history for the combination drug experiments. All model simulations were rapidly performed as finite difference calculations with an Excel 2010 spreadsheet, decreasing time steps until the resulting values were unchanged.

**Figure 2:**
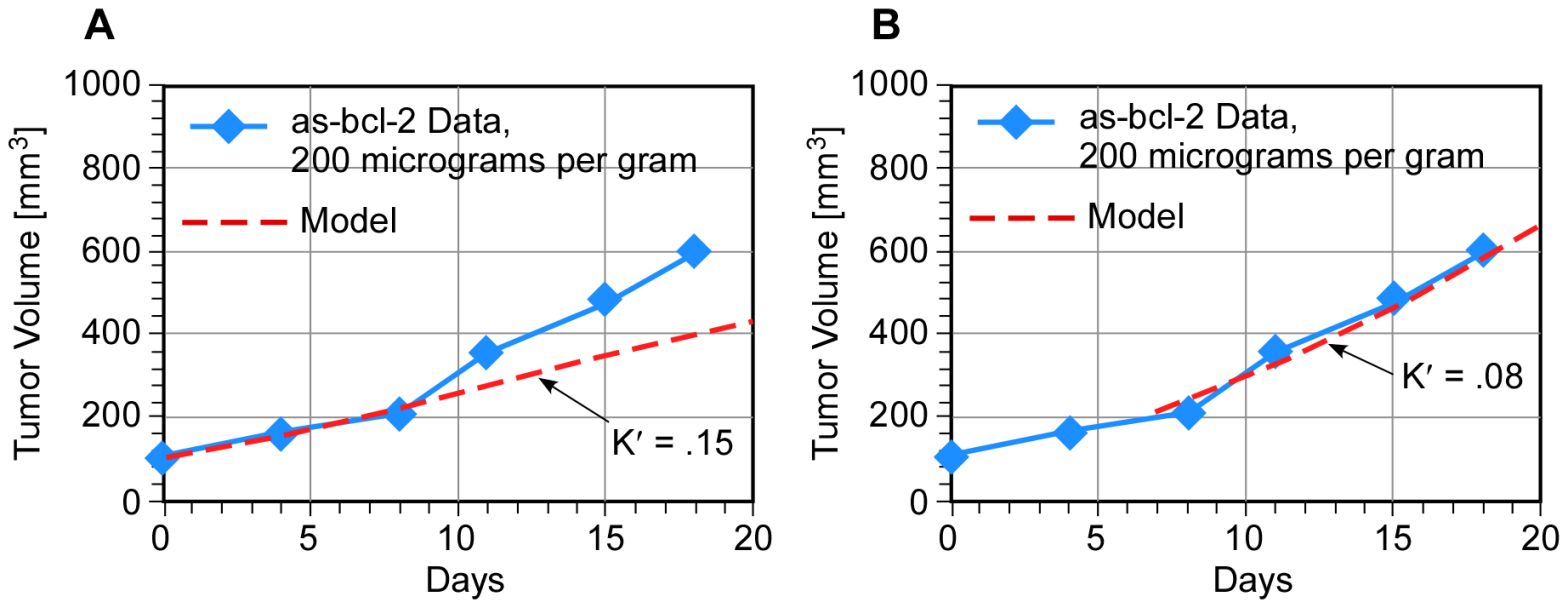
SCID mouse tumor treated with as-bcl-2 compared to (A) early time (K′ = 0.15) and (B) late time (K′ = 0.08) model.

Tumor response in these experiments was predicted with greater accuracy by subdividing the experimental period into early and late times. For example, the mean tumor volume data from the as-bcl-2 experiments (SEMs omitted for clarity) and model calculations are shown in Figure 2A (early time) and Figure 2B (late time). A reasonable explanation for this behavior is that the drugs administered at day zero were mostly depleted after 7 days. In all cases, the characteristic e-folding time for malignant cell growth was also determined from the control data to be 7 days. The late time value of K′ is virtually identical to that derived from control (no drug therapy) experiments, as would be expected if the drug is depleted.

From the early time data analysis, we determined that combination therapy increased the malignant cell death rate by a multiple of the increases for each mono-therapy, suggesting that the model captured the (early time) synergistic effects of combination therapy.

## Immune Response Calculations

**Figure 3:**
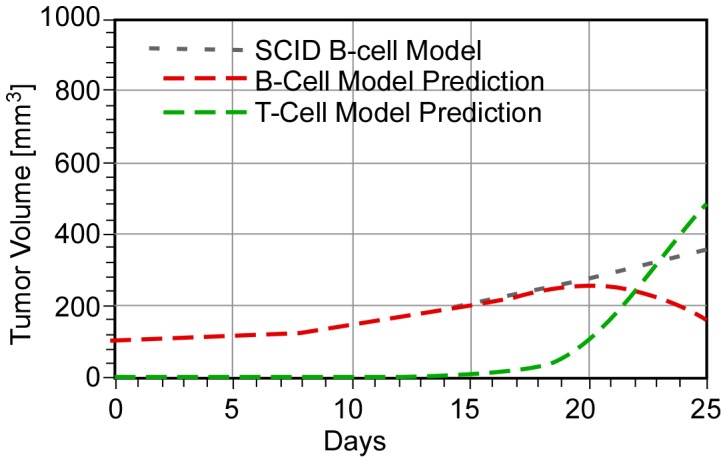
Predicted response of an immune-competent mouse to combination therapy (K″ = 4, K″′ = 1, g = 1, and B = 8).

We now use the values of K*_B_, K′ and K″ derived in Ref. 1 in a combination therapy prediction (as-bcl-2 combined with anti-CD-20) for a ***hypothetical*** immune-competent animal. Varying parameter B (for a given value of K″′), we find that ***T-cell birth rates would have to be at least eight times B-cell birth rates (B>8) for tumor regression to occur within the experimental period of 23 days***. The value of parameter K″′ appears to be less important, although it determines the rate of tumor regression. Figure 3 provides an illustrative example of the predicted behavior (compared to the SCID model [1]) for a particular set of parameter values. B-cell regression is predicted to start at about 20 days, and the T-cell population becomes dominant at later times.

## Optimum Combination Therapy

Eq. (1b) permits a given end state of tumor mass to be reached with an infinite number of combinations of values for K′ and g(d). We assume that K*_B_, K″, K″′ and B depend only on cell biology, and are not themselves functions of drug dosage. Of course, the tumor end state depends on these parameters as well, but they were held constant for this example.

The goal of any drug therapy is to provide maximum therapeutic benefit with minimum toxicity. ***Drug combinations offer the potential for achieving the same benefit as individually administered drugs, but at lower dosages*** *.* They may also prove to be beneficial to patients who have not responded to single drug regimens. Moreover, because drug benefit is generally a nonlinear function of drug dosage (i.e., a doubling of the value of g(d) will require more than a factor of two increase in dosage), determining minimum acceptable dosages has significant medical and cost implications.

**Figure 4:**
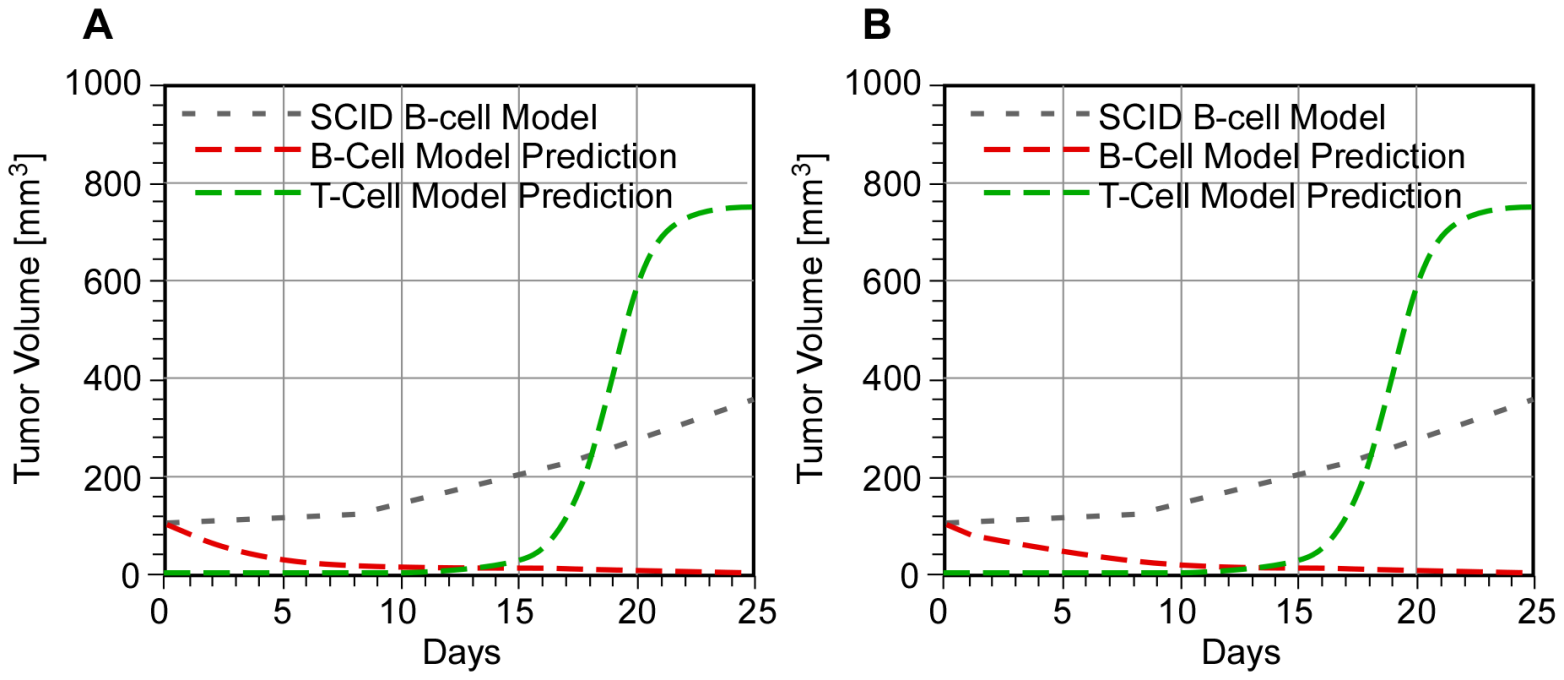
Combination as-bcl-2 and anti-CD-20 therapy to achieve N*_B_<10^−4^ in <25 days: K″ = 4, K″′ = 1 and B = 8 with (A) K′ = 0.15, g = 4 (“low-high” combination) and (B) K′ = 0.2, g = 2 (“medium-medium” combination).

Figure 4 is an example of how our parametric model can be used to find combined minimum dosages that achieve a specified level of tumor regression. Figure 4A corresponds to a “low” dose of as-bcl-2 and a “high” dose of anti-CD-20, whereas Figure 4B corresponds to a “medium” dose of each drug (both compared to the SCID model). We expect that the latter case will likely result in lower toxicity and associated side effects. The same behavior is predicted for the “high-low” combination of K′ = 0.3 and g = 1. The “medium-medium” example may not be optimum, but it suggests an approach to its determination.

## Delayed Effects

**Figure 5:**
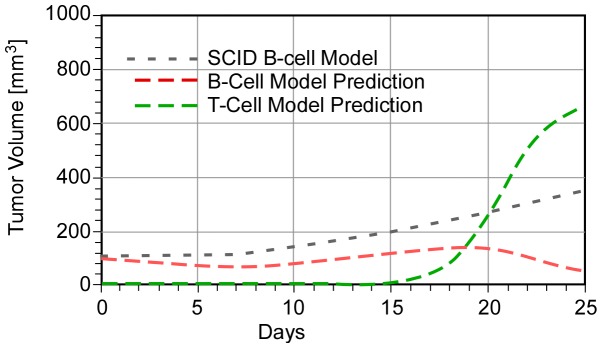
Tumor re-growth after day 7 due to depletion of as-bcl-2 (K′ = 0.25 to 0.08); regression at day 20 due to immune response (K″ = 4, K″′ = 1, g = 1 and B = 8).

Even with a strong immune response (B = 8), depletion of as-bcl-2 after 7 days results in initial re-growth of the *hypothetical* tumor, as shown in Figure 5. However, the presence of anti-CD-20 (i.e., g(d)>0) induces T-cells to kill malignant B-cells by day 20. It is implicitly assumed that this kill mechanism is neither interfering nor synergistic with direct kill by membrane penetration, but occurs in parallel with it. Similar calculations may be useful in optimizing the scheduling of drug delivery, as in the now clinically accepted use of Rituxan™ for maintenance therapy of follicular (indolent) lymphoma. [7]

## Heterogeneous Tumors

Most tumors are found to be heterogeneous, with more aggressive clones mixed with indolent strains. Even at extremely small initial populations, aggressively proliferating cells can overtake the less aggressive cells, transforming NHL from “indolent” to “aggressive” disease, typically Diffuse Large B-Cell Lymphoma [8]. This process, which is reported to occur at a rate of 2-3% per year [9], [10], [11], [12], may also be due to genetic changes in the malignant cells as the disease evolves either naturally [13] or in response to chemical or biological therapy.

Our model easily incorporates a third (or any number of) cell type with the addition of a third equation similar to Eq. (1b) for the non-dimensional population N*_a_ of an aggressive clone. Replacing N*_B_ with B-cell sub-populations N*_i_ and N*_a_, and setting N*_T_ = 0 in the SCID mouse case, we have
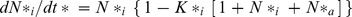
(3a)

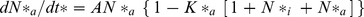
(3b)where A =  Aggressive B-cell birth rate/Indolent B-cell birth rate, K*_i_ is the same as K*_B_, and K*_a_ is the ratio of both a reduced death rate ***and*** an increased birth rate relative to normal B-cells. We expect K*_a_ to be smaller than K*_i_, resulting in larger asymptotic populations of aggressive cells as well as more rapid growth. We also recognize that within the same tumor there will be significant variations in K* among B-cells identified as members of the approximately 65 sub-groups of Non-Hodgkin Lymphoma.

**Figure 6:**
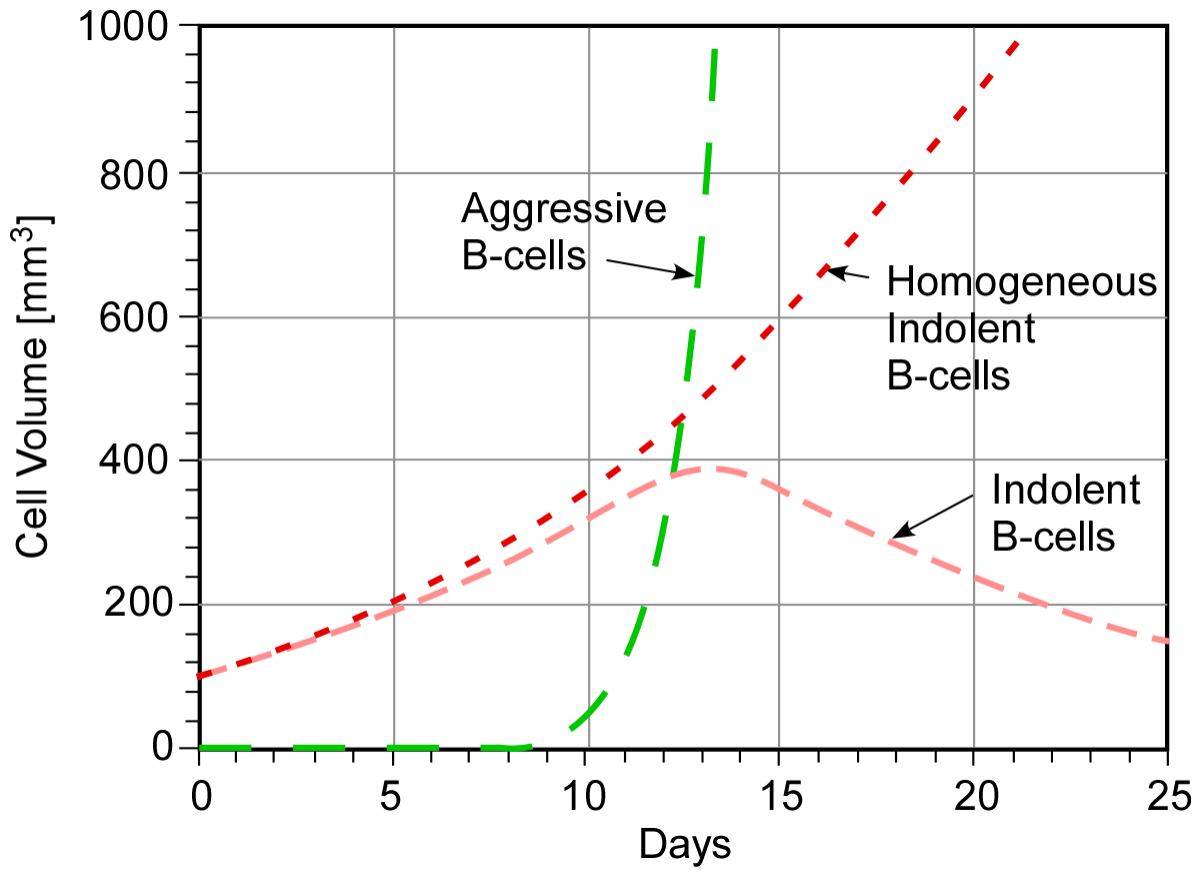
Predicted transformation of “indolent” to “aggressive” disease in a SCID animal without therapy (K*_a_ = 0.05, K*_i_ = 0.08 and A = 13).

As an illustrative example, consider the SCID case (N*_T_ = 0) without therapy. An aggressive clone population starting with an initial value of N*_a_ = 10^-6^, negligible compared to the initial population of “indolent” B-cells, is predicted in Figure 6 to dominate the indolent population by day 15. Growth of a homogeneous indolent B-cell population is plotted for comparison. Multiple model simulations indicate that for values of A, the ratio of aggressive to indolent cell birth rates, less than 12, the indolent clone remains dominant within the 25 day period shown (the corresponding occurrence of disease transformation in humans is typically many months or years). Such transformations are a serious concern for lymphoma patients over their lifetimes. The relative magnitudes of parameters A and B over the relevant time scales will be critical to this model of disease transformation in ***immune-competent*** animals and humans. We await experimental data to validate this hypothesis.

## Summary and Discussion

We have shown that our parametric model of NHL tumor response to combination therapy can be brought into excellent agreement with SCID mouse data by accounting for the apparent depletion of as-bcl-2 at later times in the experiments. The model was then extended to include the response of healthy immune (T) cells in the presence of anti-CD-20. We then predicted that a synergistic medium dosage combination of anti-CD-20 and as-bcl-2 can achieve the same level of tumor regression (10^−4^) as much higher dosages of either one of the drugs.

Delayed effects in combination therapy due to an immune response were then illustrated. This suggests that an optimum combination drug delivery schedule can be derived. Finally, we have simulated heterogeneous tumors by incorporating an aggressive cell population growth equation similar to that for indolent cells. The complex interactions of indolent and aggressive malignant cell types with healthy immune cells can also be investigated, and the conditions under which “transformation” from indolent to aggressive disease predicted.

Each of these predictions requires validation with dedicated animal experiments. The variation of each drug dose, separately and in combination, the timing of their delivery, and the extension to multiple drug combinations, would produce a rich set of experimental data with which to validate our model, as well as optimize combinations of advanced drugs.

We believe that an experimentally validated model will provide the basis for accelerated drug combination discovery in animal models, and could ultimately have significant clinical value by reducing the number and cost of human trials.
